# Supplementary material for: Dust outpaces bedrock in nutrient supply to montane forest ecosystems
Source: Nat Commun. 2017 Mar 28;8:14800. doi: 10.1038/ncomms14800 (PMC5379052; doi:10.1038/ncomms14800)
Supplement: Supplementary Information — Supplementary Figures [file ncomms14800-s1.pdf]

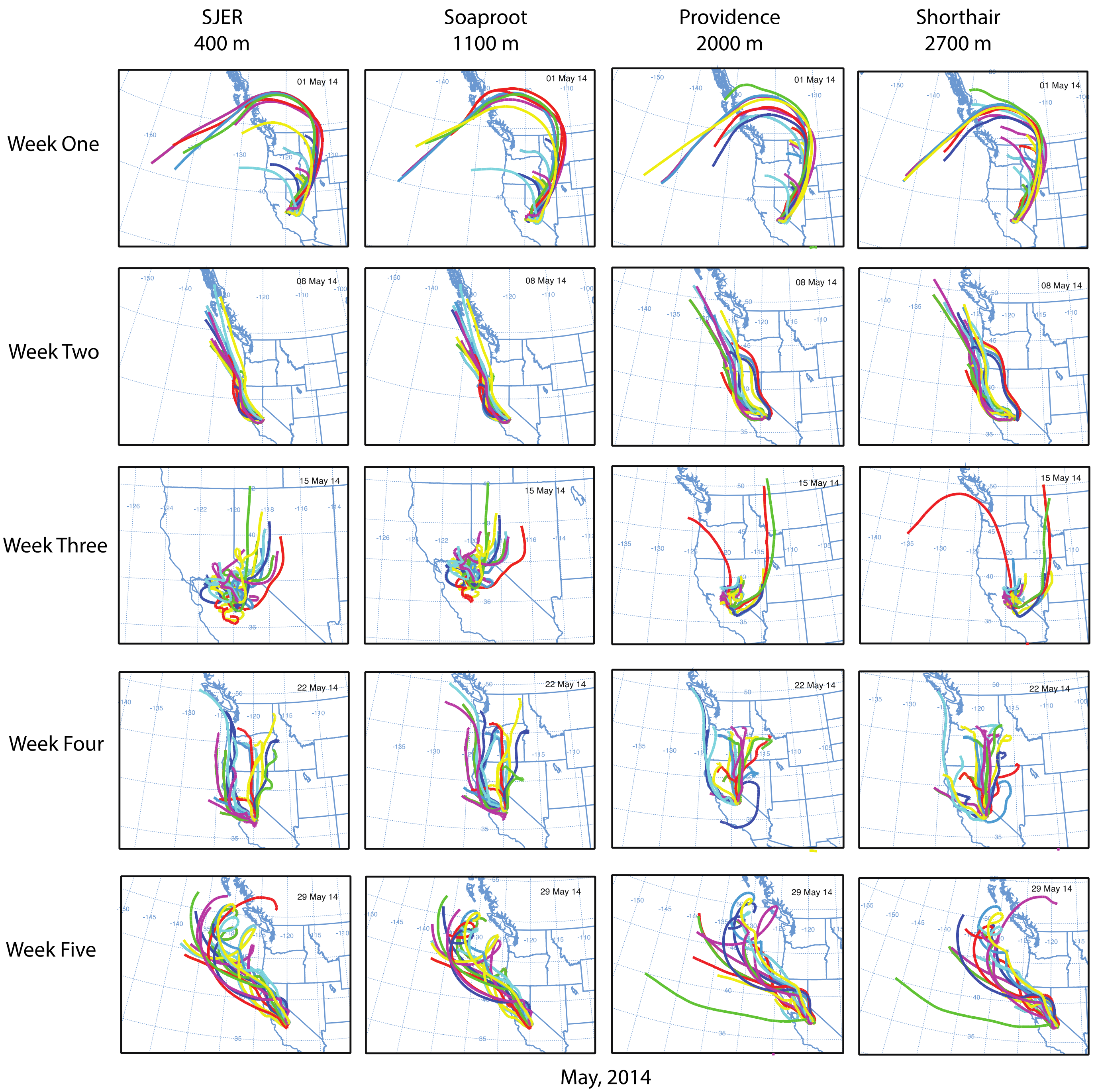

Supplementary Figure 1. Hysplit back trajectory model results for each of the four samples sites for the five weeks in May, 2014. Back trajectory tracks (different colors) are initiated every 12 hours starting 315 hours prior to 12:00 UTC time on the date on each panel. Sample sites are in columns, week of the model run in rows.

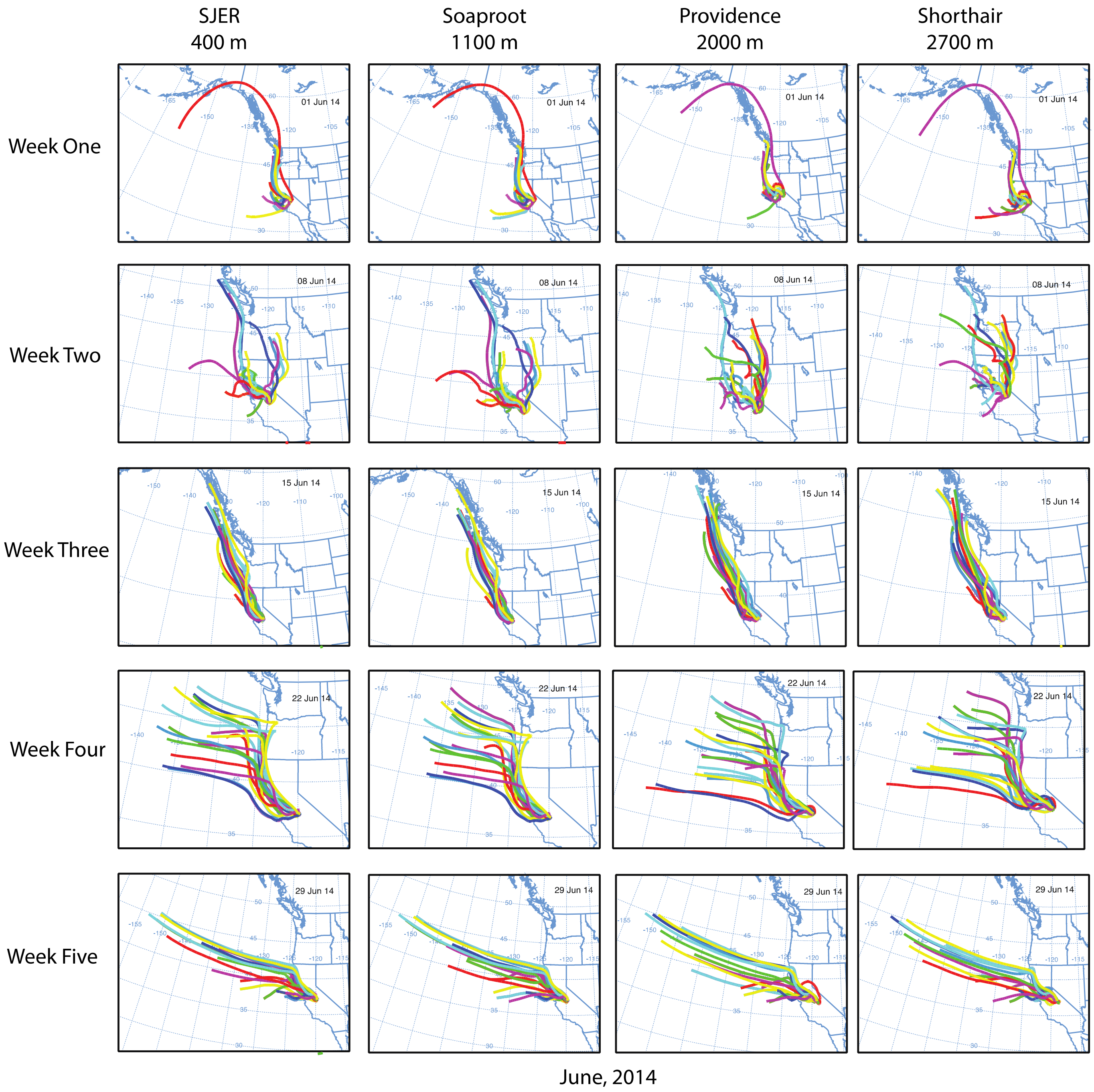

Supplementary Figure 2. Hysplit back trajectory model results for each of the four samples sites for the five weeks in June, 2014. Back trajectory tracks (different colors) are initiated every 12 hours starting 315 hours prior to 12:00 UTC time on the date on each panel. Sample sites are in columns, week of the model run in rows.

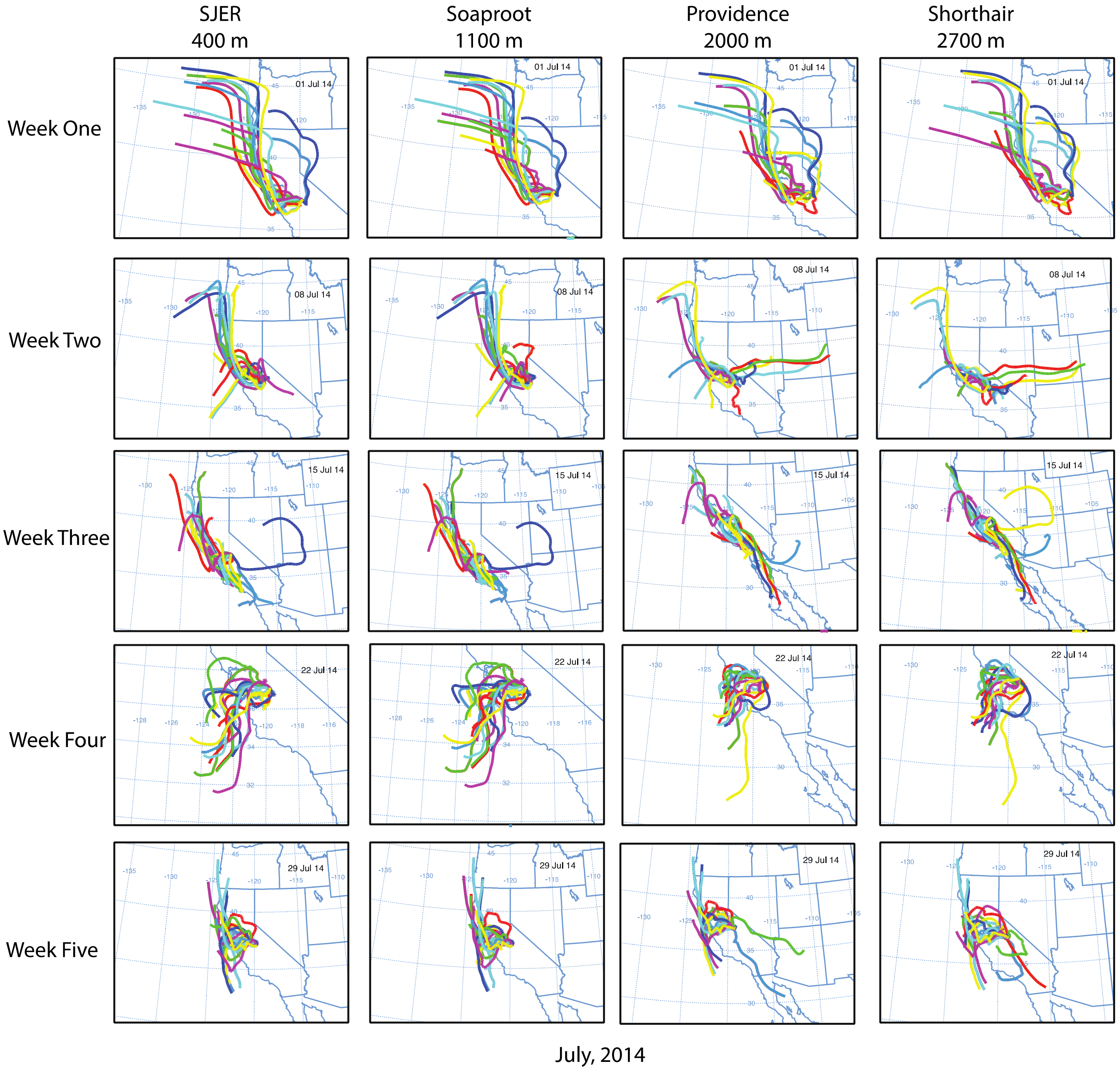

Supplementary Figure 3. Hysplit back trajectory model results for each of the four samples sites for the five weeks in July, 2014. Back trajectory tracks (different colors) are initiated every 12 hours starting 315 hours prior to 12:00 UTC time on the date on each panel. Sample sites are in columns, week of the model run in rows.

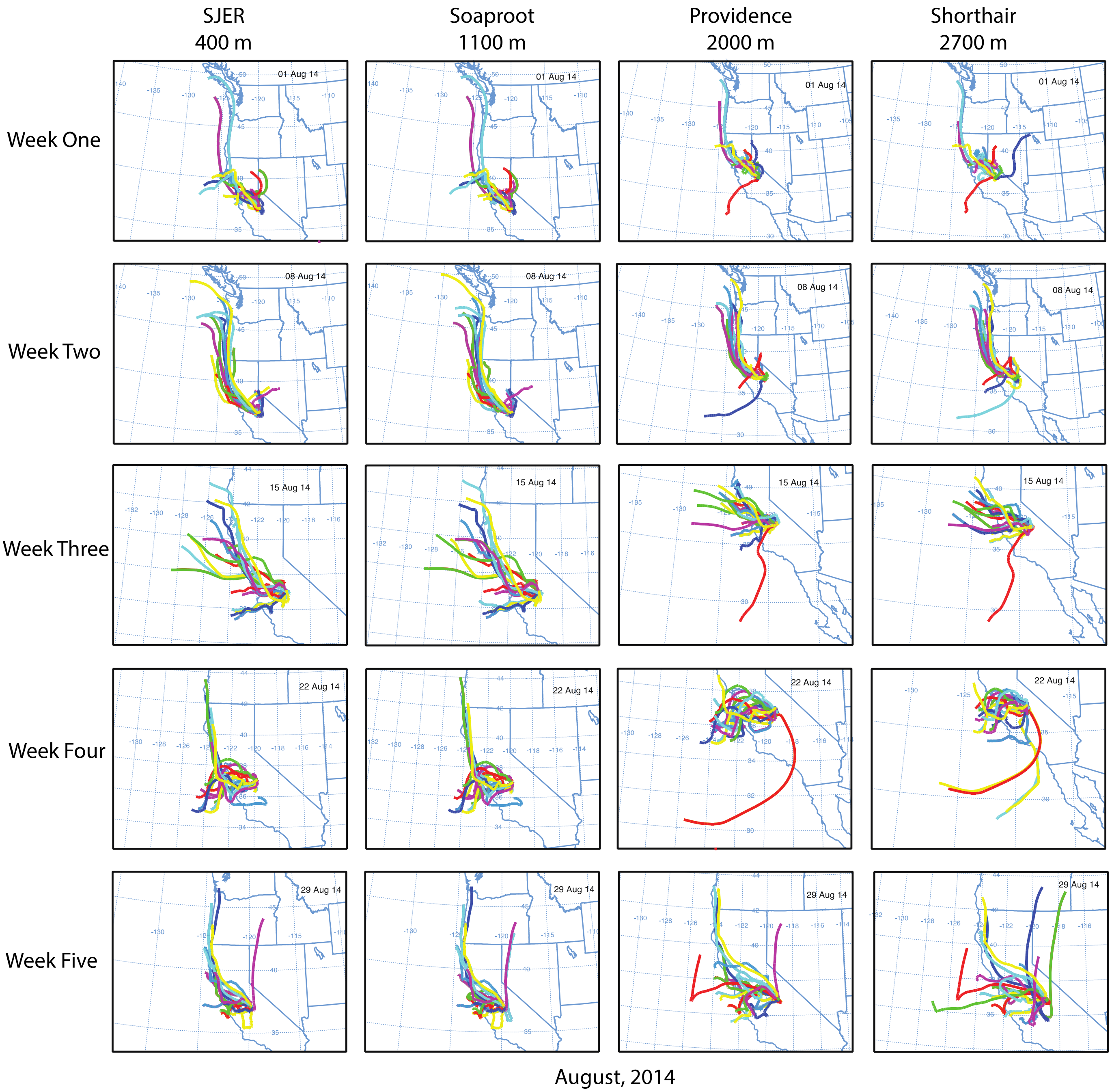

Supplementary Figure 4. Hysplit back trajectory model results for each of the four samples sites for the five weeks in August, 2014. Back trajectory tracks (different colors) are initiated every 12 hours starting 315 hours prior to 12:00 UTC time on the date on each panel. Sample sites are in columns, week of the model run in rows.
